# Remote Sensing Applications in Coastal Areas

**DOI:** 10.3390/s20092673

**Published:** 2020-05-08

**Authors:** Teodosio Lacava, Emanuele Ciancia

**Affiliations:** 1 National Research Council, Institute of Methodologies for Environmental Analysis, C. da S. Loja, 85050 Tito Scalo, Italy; 2 School of Engineering, University of Basilicata, Via dell’Ateneo Lucano 10, 85100 Potenza, Italy; emanuele.ciancia@imaa.cnr.it

## 1. Introduction

Coastal areas are regions of remarkable relevance for humans, providing essential components for social and economic development from the local to the national scale. At the same time, growing human and environmental pressures in coastal areas have contributed significantly to altering coastal ecosystem functioning. To preserve the economic and ecological sustainability of the coastal environment, the scientific community has been pushing for the use of integrated observation systems aimed at monitoring such susceptible areas. Remote sensing data can complement traditional field measurements, ensuring almost continuous synoptic coverage with a good trade-off between spatial and temporal resolution, thus allowing for a timely characterization of coastal environment dynamics. In particular, the availability of a multitemporal historical series of remote sensing data can provide useful information on the spatiotemporal variability of hydrological (sea surface currents, river runoff/discharge), biological (phytoplankton blooms, primary productivity) and physical (temperature, salinity, and turbidity) properties of coastal waters as well as on human-induced land cover mutations (deforestation, surface urban islands). This Special Issue seeks to collect high-quality papers focused on satellite-based applications for monitoring coastal areas, continental shelves, and estuarine ecosystems. Specifically, it aims to address challenges related to assessing water quality and bathymetric coastal mapping, or to identify processes in shallow and open waters, ecological threats for the ecosystem biodiversity, or rapid geomorphological or urban changes in newly developed coastal areas. All the above-mentioned topics highlight the need to develop innovative methodologies of data analysis that are able to handle multimission and multisource remote sensing data, fostering the implementation of integrated and sustainable approaches. The contents and findings of each paper published in the Special Issue on “Remote Sensing Applications in Coastal Areas” are briefly summarized in the following section.

## 2. Contributions

Mao et al. [1] investigated the spatiotemporal invasion of the exotic Spartina alterniflora (S. alterniflora) over the coast of mainland China through a multitemporal analysis of Landsat images. They quantified the areal changes of S. alterniflora from 1990 to 2015 at decade scale by using a change detection method. S. alterniflora recorded the most rapid expansion in the 2010–2015 period at a rate of 2304 ha∙yr^−1^, especially over the four central provinces of the study area. These results are useful for monitoring and management programs aimed at reducing the expansion rates of this invasive plant in China.

Wei et al. [2] evaluated the accuracy of the Global Positioning System (GPS) Kinematic Precise Point Positioning (KPPP) approach at estimating the vertical Ocean Tide Loading (OTL) displacements. They used this methodology to compute four semidiurnal (i.e., M2, S2, N2 and K2) and diurnal (i.e., K1, O1, P1 and Q1) OTL components by exploiting 12 GPS reference stations in Hong Kong (North coast of the South China Sea). Based on a 10-year analysis (2008–2017) of GPS data, the uncertainty of the GPS KPPP estimated tidal displacement was 0.2 mm for the M2, N2, O1 and Q1 tidal components and 0.5 mm for S2. This study proves the suitability of the GPS KPPP approach in accurately estimating the Earth’s vertical tidal displacement in the M2, N2, O1, and Q1 tidal frequencies.

Joliff et al. [3] discussed the potentiality of GOES-ABI data in observing ocean submesoscale processes. They developed a colorimetry approach, Chromatic Domain Mapping (CDM), aimed at convolving ocean reflectance data from polar-orbiting sensors (i.e., VIIRS, OLCI) with spectrally limited geostationary sensor data (GEOS-ABI). The CDM technique was used to successfully detect high-frequency processes that occurred in the Gulf of Mexico, such as the coastal response to Hurricane Michael. This work highlights how the joint exploitation of GOES-ABI data and the CDM methodology can facilitate the detection and revisit time of the above-mentioned submesoscale phenomena.

Mohamadi et al. [4] described a new method for monitoring Land Surface Temperature (LST) on coastal reclaimed areas. They developed a LST normalization approach, namely, LSTn, based on the averaged value of water surface temperature. The LSTn technique was tested over reclaimed areas of Lingding Bay (Southern China) by using Landsat 5–8 data at decade scale of analysis from 1987 to 2017. The LSTn implementation allowed for the identification of pronounced differences between the temperature of impervious urban surfaces and other land cover types. This study suggests the applicability of such an approach for a more robust detection of surface urban islands in newly developed coastal area.

Xing et al. [5] proposed a depth-adaptive waveform decomposition method for airborne LiDAR bathymetry aimed at facilitating coastal water mapping in the Qilianyu Islands (Hainan Province, China). This methodology allowed for the development of two best fitting models for waveforms with different depths. Field and simulated data were used to assess the accuracy of the detected water surface and bottom positions. The proposed approach performed better than the traditional methods, recording a high signal detection rate (99.11% in shallow water and 74.64% in deep water) within a large bathymetric range (0.22–40.49 m). This work reveals how the adaptive threshold of this method can allow for the reduction of fake signals, thus improving the reliability and accuracy of the signal detection.
